# Effects of Culinary Procedures on Concentrations and Bioaccessibility of Cu, Zn, and As in Different Food Ingredients

**DOI:** 10.3390/foods12081653

**Published:** 2023-04-15

**Authors:** Canchuan Zhang, Xi Miao, Sen Du, Ting Zhang, Lizhao Chen, Yang Liu, Li Zhang

**Affiliations:** 1 Key Laboratory of Tropical Marine Bio-Resources and Ecology, Guangdong Provincial Key Laboratory of Applied Marine Biology, South China Sea Institute of Oceanology, Chinese Academy of Sciences, Guangzhou 510301, China; 2 University of Chinese Academy of Sciences, Beijing 100049, China; 3 Department of Mathematics, Pennsylvania State University-Harrisburg, Middletown, PA 17057, USA; 4 Sanya Institute of Ocean Eco-Environmental Engineering, Sanya 572025, China

**Keywords:** culinary procedures, in vitro digestion, bioaccessibility, subcellular distribution, copper, zinc, arsenic

## Abstract

Although cooked diets are the primary sources for humans to absorb trace elements, there is limited data available on the concentrations and bioaccessibility of trace elements in cooked food ingredients. This work aims to evaluate the effects of culinary procedures on the concentrations and bioaccessibility of trace elements in common food ingredients. Twelve food species from the local market were treated with four culinary procedures (boiling, steaming, baking, and frying), then the bioaccessibility of copper (Cu), zinc (Zn), and arsenic (As) were evaluated using the in vitro digestion method. The subcellular distribution of these elements was also determined using the sequential fractionation method. The results show that culinary procedures decreased the retention rate of As during cooking (100% for raw and 65–89% for cooked ingredients) and the bioaccessibility of Cu and Zn during digestion (nearly 75% for raw and 49–65% for cooked ingredients), resulting in a reduction of the total bioaccessible fraction (TBF) of Cu, Zn, and As in food ingredients. The TBF of Cu, Zn, and As in all tested food ingredients followed the order: raw (76–80%) > steaming and baking (50–62%) > boiling and frying (41–50%). The effects of culinary procedures were associated with the subcellular distribution of trace elements. As was dominantly distributed in heat-stable proteins (51–71%), which were more likely to be lost during cooking. In comparison, Cu and Zn were mainly bound to the insoluble fraction and heat-denatured proteins (60–89% and 61–94% for Cu and Zn, respectively), which become less digestible in cooked ingredients. In conclusion, these results suggest that culinary procedures reduce the absorption of Cu, Zn, and As in various food ingredients, which should be considered in the coming studies related to nutrition and risk assessment of trace elements.

## 1. Introduction

Cooked diets are the main sources for humans to absorb trace elements [1]. For example, copper (Cu) and zinc (Zn) are essential trace elements for human bodies, but excessive intake can be toxic [2]. Arsenic (As) is a toxic trace element that raises concerns related to human health [3,4]. In recent decades, environmental pollution has become more serious, which could potentially result in excessive intake of trace elements from the diet [5]. As a result, the concentrations of trace elements in food ingredients have received growing attention in recent years [6,7,8]. For instance, Olmedo et al. [7] found high levels of Zn and Cu in mussels and crustaceans from the coastal waters of South Spain. Liu et al. [9] observed high accumulations of As and Cd in seafood from Laizhou Bay, China, indicating potential adverse health effects associated with seafood consumption.

Nevertheless, measuring the total element concentrations is not sufficient to accurately evaluate their risks. Bioaccessibility testing is necessary to determine whether these elements are released from the food during digestion, allowing them to be absorbed by the gut [10,11]. In vitro digestion methods are useful tools to evaluate the bioaccessibility of elements due to their low cost and convenient operation [12]. Nowadays, the most reliable in vitro model is the physiologically-based extraction test, which involves two extractions that mimic the gastric and small intestinal stages of digestion [13]. The in vitro digestion model has been used to evaluate the bioaccessibility of elements such as Cu, Zn, As, Fe, and Se [14,15]. For instance, using the in vitro digestion model, Zheng et al. [4] found that the bioaccessibility of As was between 14% and 54% among different leafy vegetables; Feitosa et al. [16] observed that the bioaccessibility of Fe and Zn were low (about 0.2% iron and 35% zinc), but the bioaccessibility of Cu was high (about 70%) in black beans. However, most studies on the bioaccessibility of trace elements have been conducted on raw foods, even though these foods are typically consumed after various culinary procedures.

The high temperatures during culinary procedures would induce structural damage, water loss, and protein denaturation, resulting in the solubilization or leaching of elements from the ingredients [17,18]. Ulaganathan et al. [19] reported that boiling and frying significantly reduced the Cu and As concentrations in shrimp (*p <* 0.05). Hosseini et al. [20] observed that Zn concentrations in kutum roach significantly decreased after boiling (*p <* 0.05). Singhato et al. [21] found a significant reduction of Se concentrations in fish (short-bodied mackerel) after boiling and frying (*p <* 0.05). However, these studies did not detect whether culinary procedures would further affect the extraction processes of these elements in the ingredients.

In addition, the subcellular distribution of trace elements could provide valuable information about their toxicity and trophic transfer. The combination of organelles, heat-stable proteins (HSP), and heat-denatured proteins (HDP) is considered the trophically available metal (TAM), while the insoluble fraction, including cellular debris (CD) and metal-rich granules (MRG), is considered unavailable [22]. Accordingly, the subcellular distribution of trace elements may also affect their bioaccessibility. Indeed, previous studies found that the bioaccessibility of trace elements was significantly correlated with the elemental subcellular distribution in several marine fish and bivalves (*p <* 0.05) [23,24]. However, whether such a relationship holds true for other food ingredients remains unknown.

The objective of this study is to investigate the effect of culinary procedures on concentrations and bioaccessibility of Cu, Zn, and As in twelve species of food ingredients (pork, beef, chicken, prawns, squid, grass bass, sea bream, oysters, clams, potatoes, kelp, and water spinach). The food ingredients were treated with four culinary procedures, including boiling, steaming, baking, and frying. The element retention rate after cooking was detected and the element bioaccessibility was evaluated using the in vitro digestion method. Moreover, the subcellular distribution of Cu, Zn, and As in raw ingredients was determined to help explain the different behavior between elements.

## 2. Materials and Methods

### 2.1. Collection and Preparation of Food Ingredients

Twelve species of food ingredients, including pork tenderloin (*Sus scrofa domesticus*), beef tenderloin (*Bubalus bubalis*), chicken breast (*Gallus domesticus*), prawns (*Penaeus vanname*), squid (*Loligo chinensis*), grass carp (*Ctenopharyngodon idellus*), sea bream (*Acanthopagrus latus*), oysters (*Crassostrea angulata*), clams (*Ruditapes philippinarum*), potatoes (*Solanum tuberosum*), kelp (*Laminaria japonica*), and water spinach (*Ipomoea aquatica* Forsk), were purchased from local markets in Guangzhou, China.

The food ingredients were transported to the laboratory using an ice box and were processed immediately after arriving at the laboratory. Pork, beef, and chicken were cut into blocks of 1 cm^3^. The muscle of the prawn was cut into strips of 1 cm. The muscle of the squid was cut into pieces of 2 cm^2^. For grass carp and sea bream, the dorsal muscle was cut into blocks of 1 cm^3^. For oysters and clams, the soft tissue was collected. The potato was peeled and cut into blocks of 1 cm^3^. The kelp was cut into pieces of 2 cm^2^. For water spinach, only the leaves were collected. All ingredients were washed with ultrapure water and immediately treated with culinary procedures.

### 2.2. Culinary Procedures

The culinary procedures included boiling, steaming, baking, and frying. Raw food ingredients were also prepared as a control. The boiling procedure was conducted in a 1 L glass beaker with 500 mL of ultrapure water and was heated with an electrothermal furnace. The steaming procedure was carried out in an electric steamer. An electric oven was used for the baking procedure at a temperature of 140 °C. The frying process was also conducted in a 1 L glass beaker with 200 mL peanut oil heated to 180 °C. Ingredients (50 g) were treated by boiling, steaming, and baking for 10 min or frying for 3 min. The beakers and ceramics used were previously soaked with 5% (*v/v*) HNO_3_ for 24 h and rinsed with ultrapure water to avoid contamination by external elements. After removing the oil or water on the surface of the food ingredients with Kimwipes, all the samples were freeze-dried to a constant weight by a vacuum freeze dryer and then ground into powder.

### 2.3. In Vitro Digestion

In vitro digestive fluids were prepared following the method used by Ruby et al. [25], with modifications. All solutions were prepared using ultrapure water. Malic acid, citric acid, acetic acid, lactic acid, pepsin, bile acid salts, pancreatin, and α-amylase from *Aspergillus oryzae* were purchased from Sigma Aldrich (Allentown, PA, USA). Simulated salivary fluid (pH = 7) contained 100 U/mL of 1% (*w/v*) α-amylase. For gastric fluid, 1 L of ultrapure water was adjusted to a pH of 2.0 with HCl (37% *m/v*, Merck, Darmstadt, Germany), and 1.25 g of porcine pepsin, 0.5 g malic acid, 0.5 mL glacial acetic acid, and 420 μL lactic acid were added. Intestinal digestive fluid was prepared by adding porcine bile and pancreatin to the test ingredients at a mass ratio of food matrices to bile and pancreatin of 1:0.175 and 1:0.05, respectively. The blank digestion solution was also analyzed in each batch of samples.

The in vitro digestion was conducted as an oral-gastric-intestinal digestion. In the oral digestive stage, 1 g of freeze-dried powder of the ingredients was mixed with 1 mL of simulated salivary fluid (pH = 7) and kept at 37 °C for 2 min. During the gastric digestion stage, 100 mL of gastric solution was added to the mixture of saliva and ingredients. All aliquots were incubated at 37 °C in a shaking incubator at 150 rpm for 1 h. For intestinal digestion, the pH was adjusted to 5.3 with saturated sodium bicarbonate (CNW, Shanghai, China). Porcine bile and pancreatin were added, and the pH was adjusted to 7.0 with 1 M NaOH (CNW, Shanghai, China). Then, the mix was incubated at 37 °C in a shaking incubator at 150 rpm. After 2 h of intestinal digestion, 2 mL of gastrointestinal fluid was sampled and centrifuged at 10,000× *g* for 10 min to separate the aqueous phase from residual materials. The supernatant was filtered through a 0.22 μm cellulose acetate disk filter and was prepared for element detection.

### 2.4. Subcellular Distribution

The subcellular distribution of Cu, Zn, and As in raw food ingredients was measured using the sequential fractionation method described by Wallace and Luoma [26]. Five different fractions were obtained altogether, including the cellular debris (CD), metal-rich granules (MRG), organelles, heat-denatured proteins (HDP), and heat-stable proteins (HSP). The detailed process is shown in the Supporting Information.

### 2.5. Measurement of Cu, Zn, and As Concentrations

The raw and cooked food ingredients, gastrointestinal fluid, and different subcellular fractions were digested using concentrated HNO_3_ (65%, trace metal grade, CNW) at 80 °C until a clear solution was obtained. After heating, the solution was diluted to an acidity of less than 5% with ultrapure water. Cu, Zn, and As in all samples were quantified by inductively coupled plasma-mass spectroscopy (ICP-MS, NexION 350X, PerkinElmer Inc., Waltham, MA, USA). An internal standard (^45^Sc for Cu, ^72^Ge for Zn and As) was selected to correct the sensitivity drift and matrix effects. The certified reference material of mussels (GBW08571) was used for quality control, with concentrations of 7.7 ± 0.9 μg g^−1^, 138 ± 9.0 μg g^−1^ and 6.1 ± 1.1 μg g^−1^ for Cu, Zn, and As, respectively. Quality control samples were analyzed every 20 samples. The actual measurements of Cu, Zn, and As in the certified reference were 7.4 ± 0.7 μg g^−1^, 139 ± 7 μg g^−1^, and 6.3 ± 1.2 μg g^−1^, respectively.

### 2.6. Calculation of the Retention Rate and Bioaccessibility

The retention rate (%) during culinary procedures was calculated by the following equation:

$$\mathrm{retention}\;\mathrm{rate}\;\left(\%\right)=\frac{C_{\mathrm{cook}}}{C_{\mathrm{raw}}}\;\times 100\%$$

where C_cook_ is the concentration of the elements (dry weight, dw) in the cooked ingredients, C_raw_ is the concentration of the elements (dw) in the raw ingredients. For raw ingredients, the retention rate was 100%.

The bioaccessibility (%) during the in vitro digestion was calculated by the following equation:

$$\mathrm{bioaccessibility}\;(\%)=\frac{C_{\mathrm{gastrointestinal}\;\mathrm{fluid}}}{C_{\mathrm{cooked}}}\;\times 100\%$$

where C_gastrointestinal fluid_ is the concentration of the elements in the gastrointestinal fluid. For raw ingredients, C_cooked_ = C_raw_.

The total bioaccessible fraction (TBF %) was calculated by the following equation:

$$\mathrm{TBF}\;(\%)=\frac{C_{\mathrm{gastrointestinal}\;\mathrm{fluid}}}{C_{\mathrm{raw}}}\;\times 100\%$$

Therefore, the TBF is also calculated by the following equation:

TBF=retention rate × bioaccessibility

### 2.7. Nutrition and Risk Assessment

The recommended dietary allowance (RDA) contribution was applied to the nutrition assessment, and hazard quotients (HQ) were applied to the risk assessment. The estimated daily intake (EDI) was calculated using the following equation:

$$\mathrm{EDI}=\frac{C_{\mathrm{sample}}\times B_{\mathrm{sample}}\times S_{\mathrm{consumption}}}{\mathrm{bw}}$$

where C_sample_ is the element concentrations in the food ingredients (μg g^−1^ wet weight); B_sample_ is the TBF of the elements in the food ingredients; S_consumption_ is the consumption of food (assuming the daily consumption of ingredients was 200 g, which was report in previous study) [27]; and bw is the average body weight of Chinese people (61.2 kg) [28]. C_sample_ was converted to the wet weight based on the moisture of the raw samples.

The RDA contribution of Cu and Zn were calculated using the following equation:

$$\mathrm{RDA}\;\mathrm{contribution}=\frac{\mathrm{EDI}\times \mathrm{bw}\;}{\mathrm{RDA}}\times 100\%$$

where RDA is the recommended dietary allowance for adults over 19 years of age, established by the NIH [29,30].

The concentrations of inorganic As but not total As were used in the HQ calculation. The percentage of inorganic arsenic in food ingredients was obtained in previous studies [31,32].

HQ of Cu, Zn, and inorganic As was calculated using the following equations:

$$\mathrm{HQ}=\frac{\mathrm{EDI}}{\mathrm{RfD}}$$

where RfD is the reference dose of the trace elements established by the USEPA [8] An HQ < 1 indicates a low risk, whereas an HQ > 1 indicates a high risk of food consumption.

### 2.8. Statistical Analyses

The graphs were created by the GraphPad Prism software (version 9.0, GraphPad Prism Inc., San Diego, CA, USA). Statistical analysis was performed using SPSS software (version 22, IBM, Armonk, NY, USA). For each species of ingredient, the differences among treated groups were tested by the one-way analysis of variance (ANOVA) procedure followed by the least significant (LSD) test or Dunnett’s T3 test for data satisfying or not satisfying the homogeneity of variance, respectively. For the average value of all species, the differences among treated groups were tested by a paired sample t-test. The correlation analysis was performed using the Pearson correlation coefficient or Spearman correlation coefficient for data satisfying or not satisfying normal distribution. All data were expressed as the means ± standard deviation (SD). A probability level (*p* value) of less than 0.05 was considered statistically significant.

## 3. Results

### 3.1. The Concentrations and Subcellular Distribution of Cu, Zn, and As in Raw Ingredients

**Figure 1:**
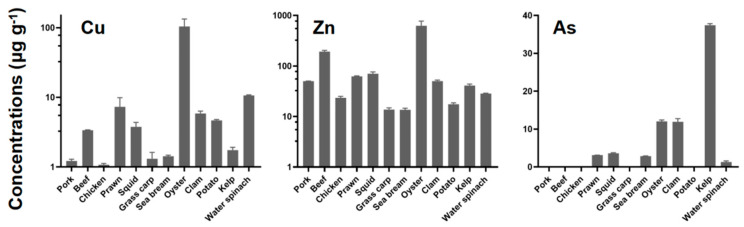
The concentrations (μg g^−1^, dry weight) of Cu, Zn, and As in the raw food ingredients. Data are expressed as means ± SD (*n* = 3).

The concentrations of Cu, Zn, and As in raw ingredients are shown in Figure 1 and Table S1. Specifically, the Cu concentrations ranged between 1.08 and 104 μg g^−1^, with oyster having the highest Cu concentrations (104 ± 29.3 μg g^−1^), followed by water spinach (10.7 ± 0.19 μg g^−1^). The Zn concentrations ranged between 13.6 and 625 μg g^−1^, with oysters and beef having higher Zn levels (625 ± 149 μg g^−1^ and 192 ± 10.8 μg g^−1^, respectively) than other ingredients (<100 μg g^−1^). Kelp had the highest concentrations of As (37.4 ± 0.41 μg g^−1^), followed by oyster and clam (12.0 ± 0.37 μg g^−1^ and 11.9 ± 0.82 μg g^−1^, respectively). However, As concentrations in pork, beef, chicken, grass carp, and potatoes were below the detection limit.

**Figure 2:**
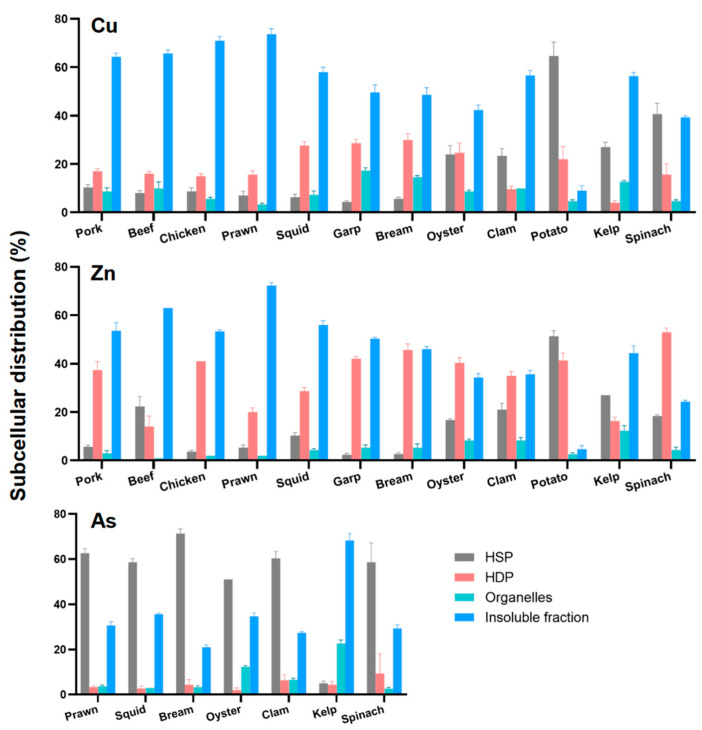
The subcellular distribution of Cu, Zn, and As in the raw food ingredients. HSP: heat-stable proteins; HDP: heat-denatured proteins; insoluble fraction: cellular debris + metal-rich granules. Data are expressed as means ± SD (*n* = 3).

Figure 2 and Table S2 indicate the subcellular distribution of Cu, Zn, and As in the raw ingredients. Except for potato and water spinach, Cu was dominantly distributed in the insoluble fraction and HDP, together accounting for 60–89% of the total Cu. For potatoes, HSP was the largest Cu pool (64%), while Cu was mainly distributed in HSP and the insoluble fraction of water spinach (79%). For most food ingredients, Zn was dominantly distributed in the HDP and insoluble fractions, together accounting for 61–94% of the total Zn. For potatoes, Zn was mainly distributed in HSP and HDP (93%). The subcellular distribution of As was clearly different from that of Cu and Zn. Except for kelp, where As was dominantly distributed in the insoluble fraction (68%), the majority of As was found in HSP for food ingredients (51–71%).

### 3.2. The Retention Rate of Cu, Zn, and As in Ingredients during Culinary Procedures

**Figure 3:**
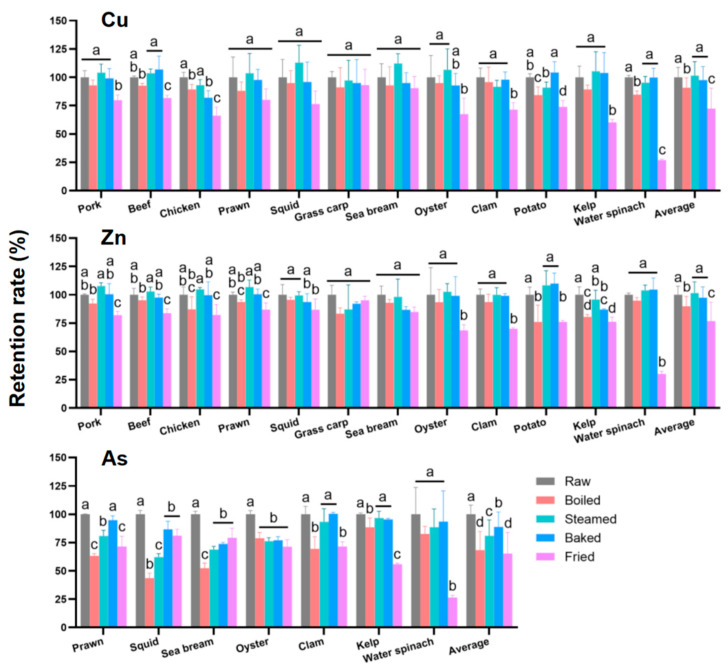
The retention rate of Cu, Zn, and As in food ingredients. Data are expressed as means ± SD (*n* = 3). Different letters (a, b, c, d) represent significant differences among treatments (*p <* 0.05).

The retention rate of Cu, Zn, and As are shown in Figure 3 and Table S3. Frying significantly decreased the retention rate of Cu and Zn (27–93% and 30–87%, respectively) in most food ingredients (*p <* 0.05), with the lowest value of Cu and Zn in water spinach. Boiling also slightly decreased the retention rate of Cu and Zn (84–96% and 76–96%, respectively). However, steaming and baking did not change the retention rate of Cu and Zn. The retention rate of As was significantly decreased by all culinary procedures (boiling: 44–88%, steaming: 62–97%, baking: 74–100%, and frying: 26–81%) (*p <* 0.05). Moreover, boiling and frying revealed a significantly lower retention rate of As than steaming and baking (*p <* 0.05). Prawns, sea bream, squid, and clams show the lowest As retention rate after boiling (63%, 44%, 52%, and 69%, respectively), while oysters, kelp, and water spinach show the lowest value after frying (71%, 56% and 26%, respectively).

In general, the average retention rate of Cu and Zn decreased significantly (72% and 77%, respectively) by frying (*p <* 0.05), slightly decreased (91% and 90%, respectively) by boiling, and was barely affected by steaming and baking (Figure 3). The average retention rate of As decreased significantly by all four cooking procedures (boiling: 68%, frying: 65%, steaming: 81% and baking: 89%) (*p <* 0.05).

### 3.3. The Bioaccessibility of Cu, Zn, and As during In Vitro Digestion

**Figure 4:**
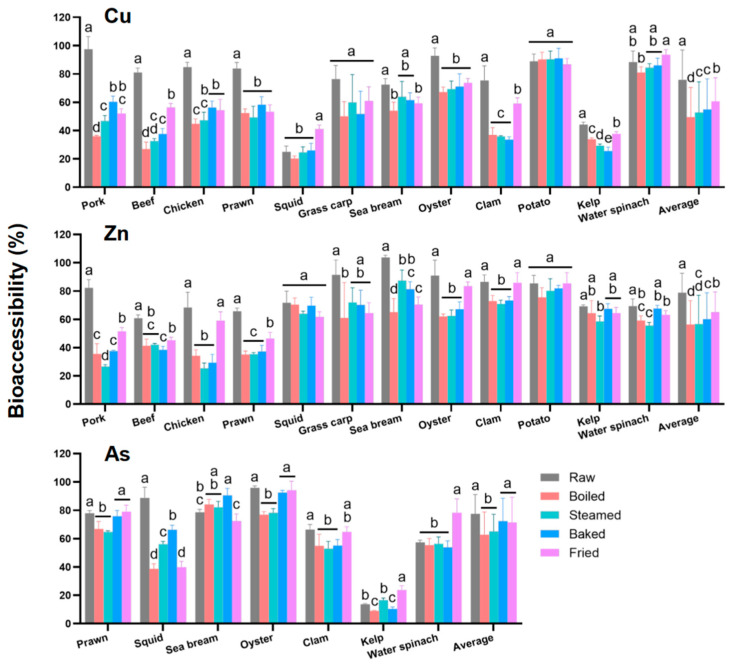
The bioaccessibility of Cu, Zn, and As in food ingredients. The kelp is not considered when calculating the average bioaccessibility of As due to its low value. Data are expressed as means ± SD (*n* = 3). Different letters (a, b, c, d) represent significant differences among treatments (*p <* 0.05).

The bioaccessibility of Cu, Zn, and As in gastrointestinal fluid is shown in Figure 4 and Table S4. Most ingredients revealed a significantly higher Cu bioaccessibility in raw (72–97%) than their cooked counterparts (27–74%) (*p <* 0.05). Squid (20–41%) and kelp (26–44%) show low Cu bioaccessibility in both raw and cooked ingredients. In contrast, potato (87–91%) and water spinach (81–94%) revealed a high Cu bioaccessibility in both raw and cooked ingredients. The results for Zn were similar to those for Cu. Raw ingredients (61–104%) revealed a higher Zn bioaccessibility than cooked ingredients (25–86%). After cooking, pork, beef, chicken, and prawns (25–59%) revealed a lower Zn bioaccessibility than other ingredients (>60%). Except for squid, there was no significant difference in As bioaccessibility between raw and cooked ingredients. The As bioaccessibility was high in oysters (77–96%) but rather low in kelp (8.9–24%).

In brief, the average bioaccessibility of Cu and Zn in cooked ingredients (49–61% and 56–65%, respectively) was significantly lower than that in raw ingredients (76% and 79%) (*p <* 0.05). No significant difference was observed in As bioaccessibility between raw, baked, and fried ingredients (77%, 72%, and 71%, respectively), while the values slightly decreased after boiling and steaming (63% and 65%, respectively).

**Figure 5:**
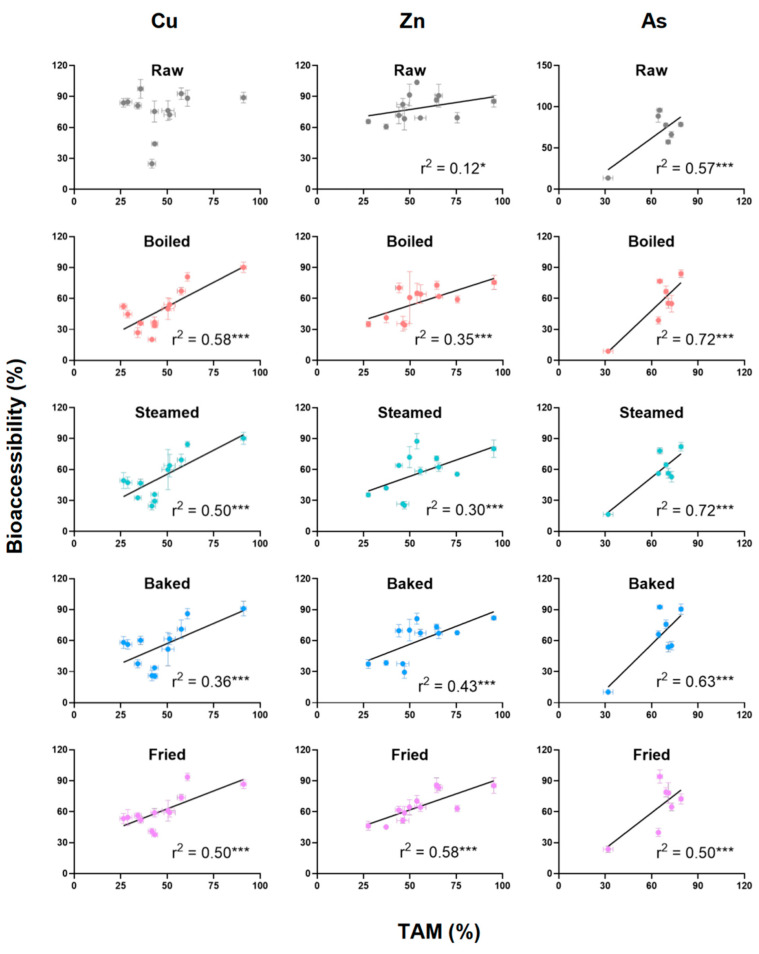
The correlation between trophically available metals (TAM) and the bioaccessibility of Cu, Zn, and As in food ingredients. TAM: organelles + HDP + HSP. The asterisk indicates there is a significant correlation (*: *p <* 0.05; ***: *p <* 0.001).

A correlation analysis was conducted to examine the relationship between trophically available metals (TAM) and the bioaccessibility of Cu, Zn, and As (Figure 5). Except for Cu in raw ingredients, there was a significant positive correlation between TAM and the bioaccessibility of Cu, Zn, and As (*p <* 0.05).

### 3.4. The TBF of Cu, Zn, and As in Food Ingredients

The total bioaccessible fraction (TBF) of Cu was high in raw ingredients (76–97%), except for squid and kelp (Figure 4 and Table S5). The culinary procedures generally decreased the TBF of Cu, with frying and boiling exhibiting a greater influence than steaming and baking. Compared with other food ingredients, squid and kelp exhibited a low TBF of Cu (19–28% and 23–44%, respectively). The TBF of Zn in raw ingredients ranged between 61 and 104%. After cooking, the TBF of Zn was largely decreased in pork, beef, chicken, and prawns (27–49%), and slightly decreased in squid, grass carp, sea bream, oysters, and clams (52–85%). The raw ingredients maintained a high TBF of As (58–96%) except for kelp. The culinary procedures decreased the TBF of As, with frying and boiling having a greater impact than steaming and baking. The TBF of As was high in oysters (60–96%) but rather low in kelp (7.8–16%).

In general, the TBF of Cu, Zn, and As in all the tested food ingredients rank in the following order: raw (76–80%) > steaming and baking (50–62%) > boiling and frying (41–50%).

### 3.5. Nutrition and Risk Assessment

Table 1 shows the recommended dietary allowance (RDA) contributions of Cu and Zn, and hazard quotients (HQ) of Cu, Zn, and inorganic As in the food ingredients (for each element, only the three ingredients with the highest concentrations were listed). The percentages of inorganic As to the total As in oysters, clams, and squid (6.3%, 2.0% and 1.7%) were obtained from previous studies [31,32]. The oysters revealed the highest bioaccessible concentrations of Cu and Zn (53.2–98.2 μg g^−1^ and 358–570 μg g^−1^, respectively) (Table S6). Consuming 200 g of oysters daily could provide 100% of the required Cu and 87% of the required Zn for humans. However, the HQ of Cu and As in oysters were 0.58–1.1 and 0.65–1.1, respectively, which exceeded the safety threshold (HQ = 1). Except for oysters, the HQ of Cu, Zn, and As in the other food ingredients were lower than the safety threshold.

## 4. Discussion

### 4.1. The Concentrations and Subcellular Distribution of Cu, Zn, and As

Among all the food ingredients tested, oysters revealed markedly higher Cu and Zn concentrations (104 ± 29 μg g^−1^ and 625 ± 149 μg g^−1^) than the other ones (Figure 1). Oysters are renowned for their excellent metal accumulation ability. In metal-polluted estuaries, oysters can accumulate Cu and Zn to rather high levels (4966 μg g^−1^ and 4897 μg g^−1^, respectively) [33], much higher than the levels observed in the present study. As was detected in only seven food ingredients, most of which were seafood. Previous studies have reported that seafood generally contains higher levels of As than food from freshwater and land, which could pose a risk to human health [34]. Kelp revealed the highest As concentrations (37 ± 0.4 μg g^−1^) in the present study. High concentrations of As (44 μg g^−1^) in seaweeds was also observed in previous studies [3], indicating that seaweeds have a strong ability to accumulation As.

The subcellular distribution was element-specific. Cu and Zn were dominantly distributed in the insoluble fraction and HDP, while As was mainly found in HSP (Figure 2). Earlier studies in marine fishes and bivalves showed that Cu and Zn were predominantly associated with the insoluble fraction, while As was mainly associated with HSP [23,24], which is consistent with the present studies. However, the Cu and Zn distribution in potatoes (mainly in HSP) and the As distribution in kelp (mainly in the insoluble fraction) were different from the other food ingredients.

### 4.2. The Retention Rate of Cu, Zn, and As

Boiling and frying decreased the Cu and Zn concentrations in the food ingredients, while steaming and baking show little change in their concentrations. (Figure 3). The decrease in Cu and Zn concentrations during boiling and frying may be due to the solubilization of these elements in the liquid medium. The results were consistent with the previous studies that boiling and frying decreased Cu and Zn concentrations in shrimp [19]. Moreover, frying had a greater influence on decreasing Cu and Zn concentrations than boiling, likely related to the different temperatures between frying and boiling (180 and 100 °C, respectively). The higher temperatures during frying could cause more damage to the structure of food ingredients, leading to a greater release of Cu and Zn from the food matrix [35]. Additionally, the different chemical compositions of oil and water may be another contributing factor. It has been reported that Cu and Zn would combine with compounds generated by chemical reactions during frying [36].

The concentrations of As decreased significantly by all four culinary procedures (*p <* 0.05). Moreover, the retention rate of As was lower than that of Cu and Zn in cooking ingredients (Figure 3). The results suggest that during cooking, As in food ingredients is more likely to be lost than Cu and Zn. This may be attributed to the varying subcellular distribution of these elements. Cu and Zn were mainly distributed in the insoluble fraction and HDP, while As was mainly distributed in HSP (Figure 2). The denaturation of proteins caused by cooking can result in the shrinkage and compaction of the insoluble fraction and HDP [23], which led to the well-preserved Cu and Zn in the food ingredients. In comparison, As in HSP was mainly in the form of small molecule compounds [3], which was easier to be lost from the food ingredients during cooking. A previous study on crustaceans and bivalves found that baking and steaming caused the loss of As from food ingredients [37]. In canned fish, the loss of As during cooking has also been found [38]. These findings were consistent with the results of the present study, suggesting that As in food ingredients can be lost during cooking.

### 4.3. The Bioaccessibility of Cu, Zn, and As

The bioaccessibility of Cu and Zn in raw ingredients (72–97%) was generally higher than that in cooked ingredients (27–74%), suggesting that culinary procedures, including boiling, steaming, baking, and frying, could reduce the bioaccessibility of Cu and Zn. However, these culinary procedures had little effect on the bioaccessibility of As, with only a slight decrease by boiling and steaming (Figure 4). Similar results have been observed in previous studies, which show a significant decrease in the bioaccessibility of Cu and Zn in shellfish, fish, and shrimp after cooking [1,23,39]. In comparison, cooking hardly changed the bioaccessibility of As in two species of fish [40]. Similarly, the bioaccessibility of As did not change in seaweeds after cooking [41,42]. Heat treatments could induce denaturation of the proteins in meat fiber, such that the tissues shrink and then become more compact, making the proteins less accessible to digestive enzymes [43]. Both Cu and Zn were mainly distributed in the insoluble fraction and HDP in the form of less easily degradable, complex, and less digestible proteins, which were more affected by heat treatments [23]. In comparison, As was mainly distributed in HSP in form of low molecular compounds [3], and the denaturation and shrinkage of protein during the thermal treatment had little effect on its bioaccessibility.

The bioaccessibility of Cu, Zn, and As also varied with the food ingredients. Potatoes and water spinach revealed a high Cu bioaccessibility in both raw and cooked ingredients (81–91%). This may be due to the high proportion of HSP-Cu in potatoes and water spinach; thus, cooking did not result in a decrease in their Cu bioaccessibility. Oysters show a high bioaccessibility of Cu, Zn, and As in both raw and cooked ingredients (>60%). High bioaccessibility of Cu and Zn (72–93%) in oysters was also observed in previous studies [44]. In contrast, the bioaccessibility of Cu in squid was low (20–41%). Squid is rich in muscle fibers and collagen, which are not easily digestible by digestive enzymes [45]. In comparison, oysters have fewer muscle fibers, resulting in more efficient digestion and higher bioaccessibility of trace elements [24]. Notably, kelp shows markedly lower As bioaccessibility (8.9–24%) than other food ingredients. Alves et al. [39] also reported that seaweeds revealed lower As bioaccessibility than other seafood, which can be attributed to the complex carbohydrate matrix structure of seaweeds that makes them difficult to digest. Our study found that As in kelp was dominantly distributed in the insoluble fraction, which might result in its low bioaccessibility. In addition, the As species may be another contributing factor. Luvonga et al. [46] reported that the majority of As species in kelp were arsenic sugar, accounting for 93% of the total As. However, arsenobetaine was found to be the predominant As species in marine animals such as fish, shrimp, squid, and oysters [47,48,49]. Laparra et al. [40] reported that the bioaccessibility of arsenobetaine in seafood (68–100%) was higher than that of other As species. Therefore, the As species in kelp might contribute to its low bioaccessibility.

The bioaccessibility of Cu and Zn in raw pork, beef, and chicken was found to be high (61–97%) but decreased markedly after cooking (27–60%). Menezes et al. [50] reported that Cu and Zn bioaccessibility in pork, beef, and chicken ranged from 8–40%, which was lower than the values in the present study. These differences in results could be attributed to variations in culinary conditions and digestion methods used in the respective studies. He et al. [23] reported that the bioaccessibility of Cu, Zn, and As in raw sea bass and sea bream was high (66–91%), and cooking markedly decreased the Cu and Zn bioaccessibility, but only slightly decreased the As bioaccessibility. Similarly, our results found that the bioaccessibility of Cu, Zn, and As in raw grass carp and sea bream ranged from 72–104%, and the Cu and Zn bioaccessibility obviously decreased but the As bioaccessibility remained unchanged after cooking. Alves et al. [39] observed that the Cu, Zn, and As bioaccessibility in raw shrimp (87%, 74% and 86%, respectively) was slightly decreased after steaming. The results were consistent with the present study, where Cu, Zn and As bioaccessibility in raw prawns (84%, 66%, and 78%, respectively) decreased after steaming (49%, 35%, and 64%, respectively).

Wallace and Luoma [26] demonstrated a significant correlation between TAM in prey and dietary assimilation by predators (*p <* 0.05), suggesting that TAM represented the bioavailable metals from the diet. Previous studies have confirmed a significant positive correlation between element bioaccessibility and TAM in marine fish, clams, and oysters (*p <* 0.05) [23,24,44]. However, previous studies have only focused on a single species or several similar species. The present study further demonstrates a significant positive correlation between element bioaccessibility and TAM across different food species (*p <* 0.05) (Figure 5), indicating that the subcellular distribution had a vital effect on the bioaccessibility of trace elements.

### 4.4. The TBF of Cu, Zn, and As in Food Ingredients

**Figure 6:**
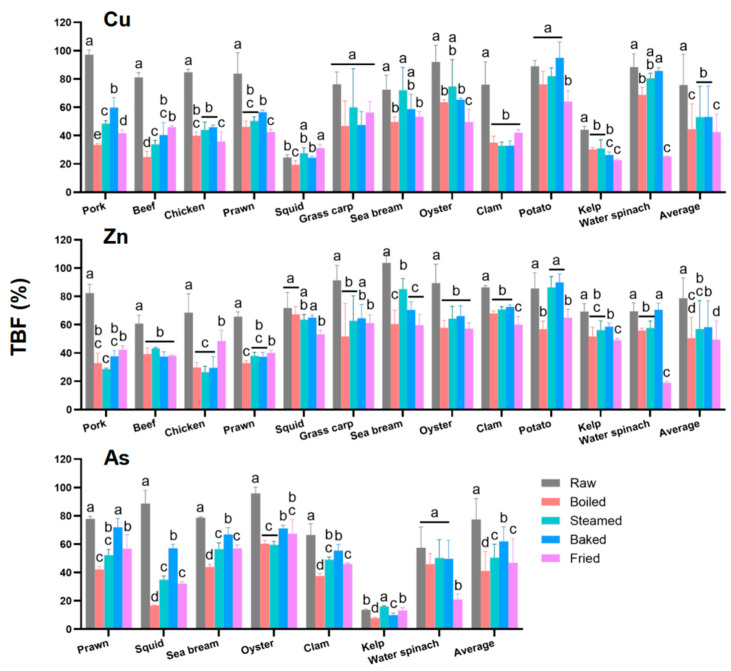
The total bioaccessible fraction (TBF) of Cu, Zn, and As in the food ingredients. The kelp is not considered when calculating the average TBF of As due to its low value. Data are expressed as means ± SD (*n* = 3). Different letters (a, b, c, d, e) represent significant differences between treatments (*p <* 0.05).

Previous studies have typically focused on the effect of culinary procedures on either element concentrations or bioaccessibility in isolation [19,39,44]. However, it is important to consider both factors simultaneously in the diet. Therefore, the present study evaluated the total bioaccessible fraction (TBF), which combines the results of retention rate and bioaccessibility, to provide a more comprehensive understanding of element uptake in food ingredients. The TBF of Cu, Zn, and As ranked in the following order: raw (76–80%) > steaming and baking (50–62%) > boiling and frying (41–50%) (Figure 6). This indicates that culinary procedures could decrease the TBF of these elements in food ingredients, with boiling and frying having a greater effect than steaming and baking. During the culinary procedures, the As retention rate was significantly reduced by cooking (65–89%) (*p <* 0.05), while the retention rates of Cu and Zn were only significantly decreased by frying (72% and 77%, respectively) (*p <* 0.05) and slightly decreased by boiling (91% and 90%, respectively) (Figure 3). During the digestion phase, the Cu and Zn bioaccessibility were significantly reduced by cooking (49–61% and 56–65%, respectively) (*p <* 0.05), while the As bioaccessibility was only slightly decreased by boiling and steaming (63% and 65%, respectively) (Figure 4). Therefore, the reduction of Cu and Zn TBF in cooked ingredients was mainly due to their low bioaccessibility. In contrast, the reduction of As TBF in cooked ingredients was mainly due to the low retention rate.

### 4.5. The Nutrition and Risk Assessments

Zn deficiency is one of the most widespread nutritional disorders, estimated to cause 1.9% of the global disease burden [2]. Therefore, it is necessary to maintain an adequate Zn intake. According to RDA contribution, oysters could provide a substantial amount of Zn required by the human body. However, an excessive Cu and As intake from oyster consumption also poses health risks to humans (HQ > 1). The culinary procedures, especially boiling and frying, could reduce the TBF of Cu and As, thus reducing the HQ of these elements in oyster. For the other food ingredients, the HQ of Cu, Zn, and As was lower than threshold (HQ = 1), suggesting that their consumption poses no health risks. Previous studies on nutrition and risk assessment have overlooked the impact of culinary procedures on the TBF of trace elements, leading to an overestimation of nutrition contributions and health risks. The present study suggests that culinary procedures could reduce the absorption of Cu, Zn, and As, which needs to be considered in future nutrition and risk assessments. In addition, it is possible to increase the absorption of essential elements and reduce the absorption of harmful ones by choosing appropriate culinary procedures.

## 5. Conclusions

The culinary procedures decreased the TBF of Cu, Zn, and As, and the TBF of these trace elements in the tested food ingredients followed the order: raw (76–80%) > steaming and baking (50–62%) > boiling and frying (41–50%). The reduction of As TBF was mainly attributed to the low retention rate during cooking (100% for raw and 65–89% for cooked ingredients), while the reduction of Cu and Zn TBF was mainly due to the low bioaccessibility during digestion (nearly 75% for raw and 49–65% for cooked ingredients). The effects of culinary procedures were associated with the subcellular distribution of trace elements. As was dominantly distributed in heat-stable proteins (51–71%), which were more likely to be lost during cooking. In comparison, Cu and Zn were mainly bound to insoluble fractions and heat-denatured proteins (60–89% and 61–94% for Cu and Zn, respectively), which become less digestible in cooked ingredients. In addition, the Cu, Zn, and As bioaccessibility varied with food ingredients. The risk assessment revealed that oyster consumption might pose potential health risks due to the excessive absorption of Cu and As, and boiling and frying should be chosen to reduce the risk of exposure to these elements. The present study provides evidence that culinary procedures can decrease the absorption of Cu, Zn, and As, highlighting the importance of considering the effects of cooking procedures in future studies related to nutrition and risk assessment of these elements.

## Figures and Tables

**Table 1:** The RDA contribution and HQ that related to food consumption.

	RDA (μg day^−1^)	RfD (μg kg^−1^ day^−1^)	Sample	BC (μg g^−1^ ww)	EDI (μg kg^−1^ day^−1^)	RDA Contribution (%)	HQ
Cu	900	40	oysters	7.1–13	23–43	157–290	0.58–1.1
			prawns	0.8–1.7	2.6–5.5	18–37	0.07–0.14
			water spinach	0.24–0.83	0.77–2.7	5.2–18	0.02–0.07
Zn	11,000	300	oysters	48–76	156–248	87–138	0.52–0.83
			beef	21–33	68–109	38–61	0.23–0.36
			squid	7.8–11	25–34	14–19	0.08–0.11
inorganic As		0.3	oysters	0.06–0.10	0.20–0.31		0.65–1.1
			clams	0.02–0.03	0.05–0.10		0.18–0.32
			kelp	0.01–0.02	0.03–0.06		0.11–0.2

RDA: recommended dietary allowance [29,30]; HQ: hazard quotient; RfD: reference doses [8]; EDI: estimated daily intake; BC: bioaccessible concentrations of trace elements.

## Data Availability

Data presented in this study are available on request from the corresponding author.

## Supplementary Materials

The following supporting information can be downloaded at: https://www.mdpi.com/article/10.3390/foods12081653/s1, Table S1: The concentrations of Cu, Zn, and As in raw food ingredients (μg g^−1^ dry weight); Table S2: The subcellular distribution of Cu, Zn, and As in raw food ingredients (%); Table S3: The retention rate of Cu, Zn, and As in food ingredients (%); Table S4: The bioaccessibility of Cu, Zn, and As in food ingredients (%); Table S5: The total bioaccessible fraction (TBF) of Cu, Zn, and As in food ingredients (%); Table S6: The total bioaccessible fraction (TBF) of Cu, Zn, and As in food ingredients (μg g^−1^ dry weight).
