# Supplementary material for: Effects of Culinary Procedures on Concentrations and Bioaccessibility of Cu, Zn, and As in Different Food Ingredients
Source: Foods. 2023 Apr 15;12(8):1653. doi: 10.3390/foods12081653 (PMC10137893; doi:10.3390/foods12081653)
Supplement: Supplementary file 1 [file foods-12-01653-s001.zip › foods-2320068-supplementary.pdf]

Supplementary Material for

Effects of culinary procedures on concentrations and bioaccessibility of Cu, Zn, and As in different food ingredients

Subcellular distribution of Cu, Zn, and As

The fresh food ingredients were frozen and ground into a power in liquid nitrogen. Approximately 1 g of food ingredient power were homogenized in 10 mL of 20 mM Tris–NaCl buffer (pH = 8.0, 5 mM antioxidant 2-mercaptoethanol, and 0.1 mM freshly prepared antiprotease phenylmethylsulfonyl fluoride). The homogenate was first centrifuged at 1500 ×*g* at 4°C for 15 min. The separated pellets were digested in 4 mL of 1 N NaOH at 80°C for 10 min and then centrifuged at 5000 ×*g* for 10 min. The secondary pellet was metal-rich granules (MRG) and the supernatant was cellular debris (CD). The supernatant from the first centrifugation was further centrifuged at 100,000 ×*g* at 4°C for 1 h. The pellet from this step was the organelles, and the supernatant was the cytosolic proteins. After heat treatment at 80°C for 10 minutes, the cytosolic proteins were cooled down in an ice bath for 30 minutes and then centrifuged at 50,000 ×g at 4°C for 30 minutes. The final pellets and supernatant were HDP and HSP fractions, respectively.

Table S1. The concentrations of Cu, Zn, and As in raw food ingredients

(μg g^{-1} dry weight)

	Cu	Zn	As
Pork	1.22 ± 0.07	50.1 ± 0.39	
Beef	3.38 ± 0.04	192 ± 10.8	
Chicken	1.08 ± 0.05	23.4 ± 1.61	
Prawns	7.32 ± 2.56	62.5 ± 1.44	3.13 ± 0.01
Squid	3.77 ± 0.60	70.3 ± 6.32	3.62 ± 0.12
Grass carp	1.31 ± 0.31	13.7 ± 1.12	
Sea bream	1.42 ± 0.05	13.6 ± 1.04	2.85 ± 0.07
Oysters	104 ± 29.3	624 ± 148	12.0 ± 0.37
Clams	5.87 ± 0.48	50.2 ± 2.56	11.9 ± 0.82
Potatoes	4.66 ± 0.15	17.52 ± 1.18	
Kelp	1.75 ± 0.17	41.1± 2.84	37.4 ± 0.41
Water spinach	10.7 ± 0.19	28.5 ± 0.43	1.31 ± 0.31

Table S2. The subcellular distribution of Cu, Zn, and As in raw food ingredients (%)

	Heat-stable proteins (HSP)	Heat-denatured proteins (HDP)	Organelle	Insoluble fraction
		Cu		
Pork	10 ± 1.2	17 ± 1.3	8.7 ± 1.2	64 ± 1.6
Beef	8.3 ± 0.8	16 ± 0.9	10 ± 2.9	66 ± 1.9
Chicken	8.6 ± 1.1	15 ± 0.8	5.4 ± 0.7	71 ± 2.2
Prawns	7.2 ± 1.6	16 ± 2.0	3.5 ± 0.2	74 ± 1.9
Squid	6.6 ± 1.3	28 ± 1.7	7.3 ± 1.8	58 ± 2.0
Grass carp	4.4 ± 0.8	29 ± 1.4	17 ± 1.5	50 ± 3.3
Sea bream	5.8 ± 0.7	30 ± 2.7	15 ± 0.6	49 ± 3.0
Oysters	24 ± 3.7	25 ± 3.6	8.6 ± 0.8	42 ± 2.2
Clams	23 ± 2.8	9.8 ± 0.9	10 ± 0.2	57 ± 2.0
Potatoes	64 ± 5.7	22 ± 5.3	4.8 ± 0.3	9 ± 1.6
Kelp	27 ± 2.2	3.8 ± 1.0	13 ± 0.5	57 ± 1.6
Water spinach	41 ± 4.9	16 ± 4.5	4.6 ± 0.2	39 ± 0.5
		Zn		
Pork	5.6 ± 0.3	38 ± 3.8	3.0 ± 1.1	54 ± 3.3
Beef	22 ± 4.0	14 ± 4.0	1.2 ± 0.1	63 ± 0.2
Chicken	3.7 ± 0.5	41 ± 0.1	1.9 ± 0.1	53 ± 0.5
Prawns	5.5 ± 1	20 ± 1.8	1.8 ± 0.1	72 ± 1.0
Squid	11 ± 1.1	29 ± 1.6	4.6 ± 0.5	56 ± 1.9
Grass carp	2.5 ± 0.3	42 ± 1.1	5.6 ± 1.1	50 ± 0.6
Sea bream	2.6 ± 0.2	46 ± 2.1	5.5 ± 1.3	46 ± 0.7
Oysters	17 ± 0.6	40 ± 2.2	8.5 ± 0.2	34 ± 1.9
Clams	21 ± 2.7	35 ± 2.0	8.3 ± 0.8	36 ± 1.0
Potatoes	52 ± 2.0	41 ± 2.7	2.6 ± 0.1	4.7 ± 1.2
Kelp	27 ± 0.3	16 ± 1.4	12 ± 1.9	44 ± 3.0
Water spinach	18 ± 0.4	53 ± 1.8	4.2 ± 0.9	25 ± 0.8
		As		
Prawns	63 ± 1.9	3 ± 0.5	3.7 ± 0.4	31 ± 1.2
Squid	59 ± 1.6	2.5 ± 1.1	3.1 ± 0.2	36 ± 0.4
Sea bream	71 ± 2.2	4.4 ± 2.0	3.2 ± 0.7	21 ± 1.3
Oysters	51 ± 0.4	2.1 ± 1.1	12 ± 0.6	35 ± 1.7
Clams	60 ± 3.0	6.2 ± 2.8	6.4 ± 0.8	27 ± 0.6
Kelp	5.1 ± 0.8	4.3 ± 1.4	22 ± 1.4	68 ± 3.2
Water spinach	59 ± 8.2	9.4 ± 8.8	2.7 ± 0.3	29 ± 1.5

Table S3. The retention rate of Cu, Zn, and As in food ingredients (%)

	raw	boiled	steamed	baked	fried
			Cu		
Pork	100 ± 5.8	93 ± 4.7	104 ± 7.4	99 ± 8.6	80 ± 4.7
Beef	100 ± 1.1	93 ± 2.0	104 ± 3.7	107 ± 12	82 ± 2.5
Chicken	100 ± 4.4	89 ± 4.4	93 ± 4.9	82 ± 6.2	66 ± 7.6
Prawns	100 ± 18	88 ± 7.9	104 ± 17	98 ± 9.3	80 ± 9.6
Squid	100 ± 16	95 ± 11	113 ± 15	96 ± 17	76 ± 12
Grass carp	100 ± 5.1	91 ± 17	97 ± 18	95 ± 21	93 ± 14
Sea bream	100 ± 12	93 ± 16	112 ± 8.5	95 ± 9	90 ± 10
Oysters	100 ± 19	95 ± 6.5	107 ± 18	93 ± 11	67 ± 14
Clams	100 ± 8.1	96 ± 13	92 ± 5.6	98 ± 6.8	71 ± 6.2
Potatoes	100 ± 3.2	84 ± 7.2	91 ± 4.9	104 ± 9.4	74 ± 5.7
Kelp	100 ± 9.8	89 ± 3.8	105 ± 17	104 ± 18	60 ± 2.7
Water spinach	100 ± 1.8	85 ± 3.3	95 ± 5.4	100 ± 8.1	27 ± 0.7
Average	100 ± 8.8	91 ± 8.7	101 ± 12	98 ± 12	72 ± 18
			Zn		
Pork	100 ± 0.8	92 ± 3.7	108 ± 2.8	100 ± 9.3	82 ± 3.3
Beef	100 ± 5.6	95 ± 2.8	103 ± 3.8	98 ± 3.0	84 ± 3.8
Chicken	100 ± 6.9	87 ± 11	105 ± 1.6	100 ± 12	82 ± 9.3
Prawns	100 ± 2.3	94 ± 1.7	107 ± 6.1	101 ± 4.4	87 ± 6.1
Squid	100 ± 9.0	96 ± 2.1	99 ± 3.2	94 ± 7.3	87 ± 9.5
Grass carp	100 ± 8.2	83 ± 5.2	87 ± 22	92 ± 1.5	95 ± 3.6
Sea bream	100 ± 7.6	93 ± 2.9	98 ± 16	87 ± 2.9	85 ± 4.3
Oysters	100 ± 24	93 ± 11	103 ± 7.1	99 ± 17	69 ± 5.1
Clams	100 ± 5.1	93 ± 7.0	100 ± 6.2	99 ± 1.9	70 ± 0.9
Potatoes	100 ± 6.7	76 ± 15	108 ± 13	110 ± 9.2	76 ± 1.3
Kelp	100 ± 6.9	80 ± 2.0	96 ± 8.1	87 ± 0.6	76 ± 4.0
Water spinach	100 ± 1.5	95 ± 2.9	104 ± 4.6	105 ± 10	30 ± 2.3
Average	100 ± 7.5	90 ± 8.6	101 ± 9.9	97 ± 9.4	77 ± 17
			As		
Prawns	100 ± 0.4	63 ± 2.0	81 ± 5.0	95 ± 3.8	71 ± 9.2
Squid	100 ± 3.4	44 ± 4.5	62 ± 3.0	87 ± 7.1	81 ± 5.7
Sea bream	100 ± 2.5	52 ± 4.5	69 ± 2.7	74 ± 1.2	79 ± 8.3
Oysters	100 ± 3.1	79 ± 5.1	76 ± 3	77 ± 3.3	71 ± 6.2
Clams	100 ± 6.9	69 ± 11	93 ± 11	100 ± 1.3	71 ± 4.3
Kelp	100 ± 1.1	88 ± 8.3	97 ± 5.8	95 ± 1.0	56 ± 1.0
Water spinach	100 ± 24	83 ± 6.6	89 ± 16	94 ± 27	26 ± 1.9
Average	100 ± 8.0	68 ± 16	81 ± 14	89 ± 13	65 ± 19

Table S4. The bioaccessibility of Cu, Zn, and As in food ingredients (%)

	raw	boiled	steamed	baked	fried
			Cu		
Pork	97 ± 9.0	36 ± 0.7	47 ± 3.9	60 ± 4.1	52 ± 3.4
Beef	81 ± 3.2	27 ± 4.8	33 ± 1.7	38 ± 3.9	56 ± 2.9
Chicken	85 ± 3.5	45 ± 3.5	47 ± 5.6	56 ± 4.6	54 ± 7.7
Prawns	84 ± 4.3	52 ± 2.9	49 ± 7.8	58 ± 5.7	53 ± 4.8
Squid	25 ± 4.2	20 ± 1.7	25 ± 3.9	26 ± 4.9	41 ± 2.8
Grass carp	76 ± 9.6	50 ± 10	60 ± 20	52 ± 16	61 ± 9.9
Sea bream	72 ± 4.3	54 ± 6.0	64 ± 11	62 ± 5.2	59 ± 4.4
Oysters	93 ± 5.6	67 ± 3.5	69 ± 5.7	71 ± 9.0	74 ± 3.0
Clams	75 ± 10	37 ± 5.2	36 ± 0.6	34 ± 2.0	59 ± 4.2
Potatoes	89 ± 5.1	90 ± 5.1	90 ± 5.8	91 ± 7.2	87 ± 4.3
Kelp	44 ± 1.9	34 ± 1.2	29 ± 1.2	26 ± 2.6	38 ± 1.4
Water spinach	88 ± 7.8	81 ± 4.1	84 ± 2.8	86 ± 5.1	94 ± 3.6
Average	76 ± 21	49 ± 21	53 ± 22	55 ± 22	61 ± 17
			Zn		
Pork	82 ± 5.8	36 ± 7.2	27 ± 1.3	38 ± 0.6	51 ± 2.6
Beef	61 ± 2.4	41 ± 4.8	42 ± 0.8	38 ± 2.4	45 ± 2.1
Chicken	68 ± 11	34 ± 4.1	25 ± 3.7	29 ± 5.9	59 ± 6.3
Prawns	66 ± 2.3	35 ± 2.6	35 ± 1.1	37 ± 4.2	46 ± 4.5
Squid	72 ± 8.2	70 ± 4.8	64 ± 1.8	70 ± 6.0	62 ± 3.8
Grass carp	91 ± 10	61 ± 25	72 ± 10	70 ± 10	64 ± 7.4
Sea bream	104 ± 1.6	65 ± 9.6	87 ± 7.4	81 ± 5.3	70 ± 5.6
Oysters	91 ± 11	62 ± 1.8	62 ± 4.2	67 ± 5.1	83 ± 3.1
Clams	87 ± 5.0	73 ± 4.0	71 ± 2.6	73 ± 2.7	86 ± 7.2
Potatoes	85 ± 5.7	76 ± 6.8	80 ± 8.5	82 ± 2.1	85 ± 7.6
Kelp	69 ± 1.1	64 ± 9.0	59 ± 3.8	67 ± 3.5	64 ± 4.1
Water spinach	69 ± 5.0	59 ± 3.4	56 ± 2.2	68 ± 2.2	63 ± 3.0
Average	79 ± 14	56 ± 17	57 ± 20	60 ± 19	65 ± 14
			As		
Prawns	78 ± 2.0	67 ± 5.3	65 ± 0.9	76 ± 4.1	79 ± 4.6
Squid	89 ± 7.5	39 ± 3.5	56 ± 1.8	66 ± 3.3	40 ± 4.1
Sea bream	79 ± 2.1	84 ± 3.6	82 ± 4.1	91 ± 5.0	72 ± 5.0
Oysters	96 ± 1.4	77 ± 2.1	78 ± 3.0	93 ± 1.6	94 ± 6.4
Clams	66 ± 3.7	55 ± 8.0	53 ± 5.1	55 ± 4.1	65 ± 3.6
Kelp	14 ± 0.5	8.9 ± 0.5	17 ± 1.5	10 ± 1.4	24 ± 3.0
Water spinach	57 ± 1.5	55 ± 4.8	56 ± 4.8	54 ± 4.6	78 ± 9.9
Average	77 ± 14	63 ± 16	65 ± 12	72 ± 16	71 ± 18

Table S5. The total bioaccessible fraction (TBF) of Cu, Zn, and As in food ingredients (%)

	raw	boiled	steamed	baked	fried
			Cu		
Pork	97 ± 3.3	33 ± 1.0	49 ± 2.1	60 ± 7.1	42 ± 2.3
Beef	81 ± 3.4	25 ± 3.9	34 ± 2.8	40 ± 8.7	46 ± 1.0
Chicken	85 ± 2.2	40 ± 2.5	44 ± 5.5	46 ± 1.5	36 ± 6.8
Prawns	84 ± 15	46 ± 4.1	50 ± 2.9	57 ± 1.5	42 ± 2.1
Squid	24 ± 2.0	19 ± 3.1	28 ± 3.7	24 ± 1.0	31 ± 2.6
Grass carp	76 ± 8.8	47 ± 18	60 ± 27	47 ± 9.5	56 ± 8.0
Sea bream	72 ± 10	50 ± 3.7	72 ± 16	59 ± 11	53 ± 3.7
Oysters	92 ± 12	64 ± 1.8	75 ± 19	65 ± 1.2	50 ± 9.1
Clams	76 ± 16	35 ± 4.5	33 ± 2.4	33 ± 3.3	42 ± 2.1
Potatoes	89 ± 4.2	76 ± 9.5	82 ± 5.9	95 ± 11	64 ± 7.5
Kelp	44 ± 2.5	30 ± 1.3	31 ± 6.1	26 ± 2.1	23 ± 0.9
Water spinach	88 ± 9.3	69 ± 5.4	80 ± 3.6	86 ± 2.3	25 ± 0.3
Average	76 ± 22	44 ± 18	53 ± 22	53 ± 22	42 ± 13
			Zn		
Pork	82 ± 6.4	33 ± 7.0	29 ± 0.6	38 ± 4	42 ± 2.9
Beef	61 ± 5.9	39 ± 4.3	43 ± 0.8	38 ± 3.4	38 ± 0.8
Chicken	69 ± 13	30 ± 3.6	27 ± 4.1	29 ± 7.9	48 ± 7.7
Prawns	66 ± 3.3	33 ± 1.8	38 ± 2.6	37 ± 3	40 ± 2.0
Squid	72 ± 11	67 ± 5.5	64 ± 3.6	65 ± 1.8	53 ± 2.8
Grass carp	91 ± 11	52 ± 23	63 ± 18	65 ± 9.6	61 ± 5.9
Sea bream	104 ± 6.7	60 ± 9.8	85 ± 7.4	70 ± 5.7	60 ± 7.5
Oysters	89 ± 13	58 ± 5.3	64 ± 8.7	66 ± 7.2	57 ± 4.1
Clams	86 ± 1.3	68 ± 1.6	71 ± 2.0	73 ± 1.4	60 ± 5.8
Potatoes	86 ± 11	57 ± 5.7	86 ± 7.7	90 ± 6.0	65 ± 6.0
Kelp	69 ± 5.8	52 ± 6.6	56 ± 6.6	59 ± 2.6	49 ± 1.2
Water spinach	69 ± 6.0	56 ± 1.5	58 ± 4.9	71 ± 4.7	19 ± 1.1
Average	79 ± 14	50 ± 15	57 ± 20	58 ± 19	49 ± 13
			As		
Prawns	78 ± 1.8	42 ± 2.3	52 ± 4	72 ± 6.2	57 ± 10
Squid	89 ± 9.2	17 ± 0.3	35 ± 2.7	57 ± 2.7	32 ± 1.4
Sea bream	79 ± 0.7	44 ± 2	57 ± 4.4	67 ± 4.7	57 ± 2.3
Oysters	96 ± 4.3	60 ± 2.3	60 ± 2.4	71 ± 2.1	67 ± 9.9
Clams	66 ± 8	38 ± 1.7	49 ± 1.9	55 ± 4.4	46 ± 0.7
Kelp	14 ± 0.5	7.8 ± 0.5	16 ± 0.6	9.9 ± 1.4	13 ± 1.8
Water spinach	57 ± 14	46 ± 7.5	50 ± 13	50 ± 13	21 ± 4.1
Average	77 ± 15	41 ± 14	50 ± 9.5	62 ± 10	47 ± 17

Table S6. The total bioaccessible fraction (TBF) of Cu, Zn, and As in food ingredients ($\mu g\ g^{-1}$ dry weight)

	raw	boiled	steamed	baked	fried
			Cu		
Pork	1.18 ± 0.04^{a}	0.41 ± 0.01^{b}	0.59 ± 0.06^{c}	0.73 ± 0.05^{d}	0.51 ± 0.01^{e}
Beef	2.74 ± 0.13^{a}	0.84 ± 0.13^{b}	1.14 ± 0.11^{c}	1.37 ± 0.29^{cd}	1.55 ± 0.04^{d}
Chicken	0.91 ± 0.05^{a}	0.43 ± 0.01^{bc}	0.47 ± 0.04^{b}	0.49 ± 0.03^{b}	0.38 ± 0.06^{c}
Prawns	6.38 ± 3.42^{a}	3.43 ± 1.47^{bc}	3.64 ± 1.04^{bc}	4.12 ± 1.33^{b}	3.07 ± 0.91^{c}
Squid	0.92 ± 0.19^{a}	0.74 ± 0.22^{b}	1.05 ± 0.28^{ac}	0.92 ± 0.17^{a}	1.18 ± 0.25^{c}
Grass carp	1.00 ± 0.25^{a}	0.58 ± 0.10^{a}	0.82 ± 0.53^{a}	0.64 ± 0.28^{a}	0.75 ± 0.28^{a}
Sea bream	1.03 ± 0.18^{a}	0.70 ± 0.03^{b}	1.03 ± 0.23^{a}	0.84 ± 0.18^{ab}	0.76 ± 0.05^{b}
Oysters	98.2 ± 41.2^{a}	66.5 ± 20.4^{b}	75.0 ± 11.9^{ab}	68.1 ± 18.7^{b}	53.2 ± 24.0^{c}
Clams	4.51 ± 1.30^{a}	2.07 ± 0.42^{b}	1.93 ± 0.09^{b}	1.93 ± 0.21^{b}	2.46 ± 0.11^{b}
Potatoes	4.14 ± 0.23^{a}	3.55 ± 0.55^{a}	3.82 ± 0.22^{a}	4.43 ± 0.66^{a}	2.99 ± 0.37^{b}
Kelp	0.77 ± 0.12^{a}	0.53 ± 0.07^{b}	0.54 ± 0.11^{b}	0.46 ± 0.07^{bc}	0.40 ± 0.02^{c}
Water spinach	9.43 ± 1.16^{a}	7.32 ± 0.69^{b}	8.57 ± 0.53^{ab}	9.13 ± 0.39^{a}	2.68 ± 0.01^{c}
			Zn		
Pork	41.3 ± 3.5^{a}	16.5 ± 3.5^{bc}	14.4 ± 0.2^{c}	18.9 ± 2.2^{bc}	21.2 ± 1.4^{b}
Beef	117 ± 18^{a}	75.8 ± 12.2^{b}	83.3 ± 3.7^{b}	72.4 ± 9.6^{b}	72.9 ± 3.5^{b}
Chicken	16.2 ± 4.0^{a}	6.98 ± 1.34^{b}	6.26 ± 1.40^{b}	6.89 ± 1.79^{b}	11.4 ± 2.3^{c}
Prawns	41.1 ± 2.9^{a}	20.5 ± 0.8^{b}	23.6 ± 1.3^{bc}	23.3 ± 1.5^{bc}	25.0 ± 0.7^{c}
Squid	50.8 ± 11.7^{a}	47.5 ± 8.3^{a}	44.5 ± 1.4^{ab}	45.7 ± 5.4^{a}	37.5 ± 5.0^{b}
Grass carp	12.6 ± 2.0^{a}	7.23 ± 3.7^{b}	8.74 ± 3.09^{b}	8.79 ± 0.62^{ab}	8.42 ± 1.50^{b}
Sea bream	14.1 ± 2.0^{a}	8.19 ± 1.06^{b}	11.6 ± 1.9^{c}	9.62 ± 1.52^{b}	8.08 ± 0.51^{b}
Oysters	570 ± 208^{a}	358 ± 66^{b}	396 ± 67^{b}	419 ± 143^{b}	360 ± 109^{b}
Clams	43.3 ± 2.0^{a}	34.1 ± 2.1^{b}	35.5 ± 2.8^{b}	36.5 ± 2.5^{b}	30.1 ± 3.4^{c}
Potatoes	15.1 ± 3.0^{a}	9.90 ± 1.07^{b}	15.1 ± 1.4^{a}	15.8 ± 1.5^{a}	11.3 ± 0.6^{b}
Kelp	28.5 ± 4.4^{a}	21.2 ± 2.6^{bc}	23.1 ± 3.44^{bc}	24.0 ± 1.0^{b}	20.1 ± 1.3^{c}
Water spinach	19.8 ± 2^{a}	15.9 ± 0.6^{b}	16.5 ± 1.5^{b}	20.1 ± 1.1^{c}	5.41 ± 0.34^{d}
			As		
Prawns	2.43 ± 0.05^{a}	1.32 ± 0.07^{b}	1.63 ± 0.12^{bc}	2.25 ± 0.20^{a}	1.77 ± 0.31^{c}
Squid	3.22 ± 0.33^{a}	0.61 ± 0.01^{d}	1.26 ± 0.10^{c}	2.07 ± 0.10^{d}	1.17 ± 0.05^{c}
Sea bream	2.24 ± 0.02^{a}	1.25 ± 0.06^{d}	1.61 ± 0.12^{c}	1.91 ± 0.13^{d}	1.63 ± 0.06^{c}
Oysters	11.5 ± 0.5^{a}	7.27 ± 0.27^{b}	7.17 ± 0.29^{b}	8.57 ± 0.25^{c}	8.08 ± 0.95^{bc}
Clams	7.97 ± 1.46^{a}	4.48 ± 0.21^{b}	5.85 ± 0.23^{cd}	6.61 ± 0.53^{c}	5.49 ± 0.40^{d}
Kelp	5.1 ± 0.2^{a}	2.93 ± 0.24^{b}	5.98 ± 0.29^{c}	3.72 ± 0.59^{d}	4.96 ± 0.71^{a}
Water spinach	0.78 ± 0.39^{a}	0.60 ± 0.14^{a}	0.68 ± 0.35^{a}	0.68 ± 0.33^{a}	0.27 ± 0.05^{b}

Different letters (a, b, c, d, e) represent significant differences between different treatment groups ($p < 0.05$)
